# Supplementary material for: Insect size responses to climate change vary across elevations according to seasonal timing
Source: PLoS Biol. 2025 Jan 30;23(1):e3002805. doi: 10.1371/journal.pbio.3002805 (PMC11783300; doi:10.1371/journal.pbio.3002805)
Supplement: S1 Text — Table A. ANOVA results examining how the body size anomaly varies over time (historic or current), with the summer temperature anomaly (Tsum), and with time and the summer temperature anomaly. We additionally account for elevation (elev) and seasonal timing (SpTiming: nymphal diapauser, early season, late season). We report model Akaike and Bayesian information criterion (AIC and BIC, respectively), where lower values indicate better model performance. Table B. ANOVA results examining how the body size anomaly varies over time (historic or current), with the developmental temperature anomaly (Tdev), and with time and the developmental temperature anomaly. We additionally account for elevation (elev) and seasonal timing (SpTiming: nymphal diapauser, early season, late season). We report model Akaike and Bayesian information criterion (AIC and BIC, respectively), where lower values indicate better model performance. Table C. ANOVA examining changes in the body size anomaly in response to phenology anomaly, elevation (elev), sex, and species. Fig A. Body size (femur length, mm) declines with elevation, females are substantially larger than males, and the body size clines vary with seasonal timing. We depict both violin plots and population means ± SE by sex and elevation. The species are arranged by seasonal timing from the earliest season, nymphal diapausing species to late season species (left to right and top to bottom). Body size changes between the historic and current time period differ based on seasonal timing and elevation. The slope of the relationship with elevation during the historic period (accounting sex as an intercept) generally declines with later seasonal timing (slope + SE = Eritettix simplex: −0.40 ± 0.07, Xanthippus corallipes: −0.45 ± 0.22, Aeropedellus clavatus: −0.48 ± 0.05, Melanoplus boulderensis: −0.35 ± 0.11, Camnula pellucida: −0.29 ± 0.13, Melanoplus sanguinipes: −0.11 ± 0.22). The data and code needed to generate this figure can be found [file pbio.3002805.s001.pdf]

S1 Text

Table A. ANOVA results examining how the body size anomaly varies over time (historic or current), with the summer temperature anomaly (Tsum), and with time and the summer temperature anomaly. We additionally account for elevation (elev) and seasonal timing (SpTiming: nymphal diapauser, early season, late season). We report model Akaike and Bayesian information criterion (AIC and BIC, respectively), where lower values indicate better model performance.

		TIME AIC=5483, BIC=5568				TEMPERATURE AIC=5417, BIC=5502				TIME + TEMPERATURE AIC=5389, BIC=5546			
	NumDF	DenDF	F value	Pr(>F)		DenDF	F value	Pr(>F)		DenDF	F value	Pr(>F)	
Tsum	1					341.74	26.12	0	***	707.74	5.14	0.02	*
time	1	60.24	0.05	0.82						412.65	0.56	0.45	
elev	1	1134.71	0.54	0.46		1217.82	1.18	0.28		755.88	2.39	0.12	
SpTiming	2	55.78	0.19	0.83		38.92	0	1		303.72	2.79	0.06	
Tsum:time	1									707.74	10.99	0	***
Tsum:elev	1					558.91	9.56	0	**	694.24	3.5	0.06	
time:elev	1	1134.71	0.37	0.54						755.88	0.43	0.51	
Tsum:SpTiming	2					354.52	0.75	0.47		708.92	0.8	0.45	
time:SpTiming	2	55.78	0.85	0.43						303.72	0.16	0.85	
elev:SpTiming	2	1238.69	7.99	0	***	1286.5	30.59	0	***	821.41	0.92	0.4	
Tsum:time:elev	1									694.24	6.13	0.01	*
Tsum:time:SpTiming	2									708.92	4.17	0.02	*
Tsum:elev:SpTiming	2					451.35	16.43	0	***	723.73	1.86	0.16	
time:elev:SpTiming	2	1238.69	4.14	0.02	*					821.41	1.8	0.17	
Tsum:time:elev:SpTiming	2									723.73	7.61	0	***

Table B. ANOVA results examining how the body size anomaly varies over time (historic or current), with the developmental temperature anomaly (Tdev), and with time and the developmental temperature anomaly. We additionally account for elevation (elev) and seasonal timing (SpTiming: nymphal diapauser, early season, late season). We report model Akaike and Bayesian information criterion (AIC and BIC, respectively), where lower values indicate better model performance.

		TIME AIC=5483, BIC=5568				TEMPERATURE AIC=5481, BIC=5565				TIME + TEMPERATURE AIC=5472, BIC=5629			
	NumDF	DenDF	F value	Pr(>F)		DenDF	F value	Pr(>F)		DenDF	F value	Pr(>F)	
Tdev	1					171.35	2.71	0.1		454.53	0.58	0.44	
time	1	60.24	0.05	0.82						100.58	0.1	0.75	
elev	1	1134.71	0.54	0.46		934.6	0.09	0.76		755.35	0.94	0.33	
SpTiming	2	55.78	0.19	0.83		48.24	0.48	0.62		80.64	0.94	0.39	
Tdev:time	1									454.53	1.73	0.19	
Tdev:elev	1					326.3	3.22	0.07		433.98	0.02	0.88	
time:elev	1	1134.71	0.37	0.54						755.35	0.97	0.32	
Tdev:SpTiming	2					181.07	7.96	0	***	386.99	0.99	0.37	
time:SpTiming	2	55.78	0.85	0.43						80.64	0.52	0.59	
elev:SpTiming	2	1238.69	7.99	0	***	997.88	12.63	0	***	641.5	6.12	0	**
Tdev:time:elev	1									433.98	0.36	0.55	
Tdev:time:SpTiming	2									386.99	3.63	0.03	*
Tdev:elev:SpTiming	2					420.69	1.18	0.31		461.76	0.11	0.89	
time:elev:SpTiming	2	1238.69	4.14	0.02	*					641.5	3.51	0.03	*
Tdev:time:elev:SpTiming	2									461.76	0.92	0.4	

Table C. ANOVA examining changes in the body size anomaly in response to phenology anomaly, elevation (elev), sex, and species.

	NumDF	DenDF	F value	Pr(>F)	
doy	1	1781.85	20.56	0	***
elev	1	341.92	0.47	0.49	
SpTiming	2	33.42	0.36	0.7	
doy:elev	1	1749.15	0.34	0.56	
doy:SpTiming	2	1770.24	1.4	0.25	
elev:SpTiming	2	316.47	6.19	0	**
doy:elev:SpTiming	2	1741.24	0.33	0.72	

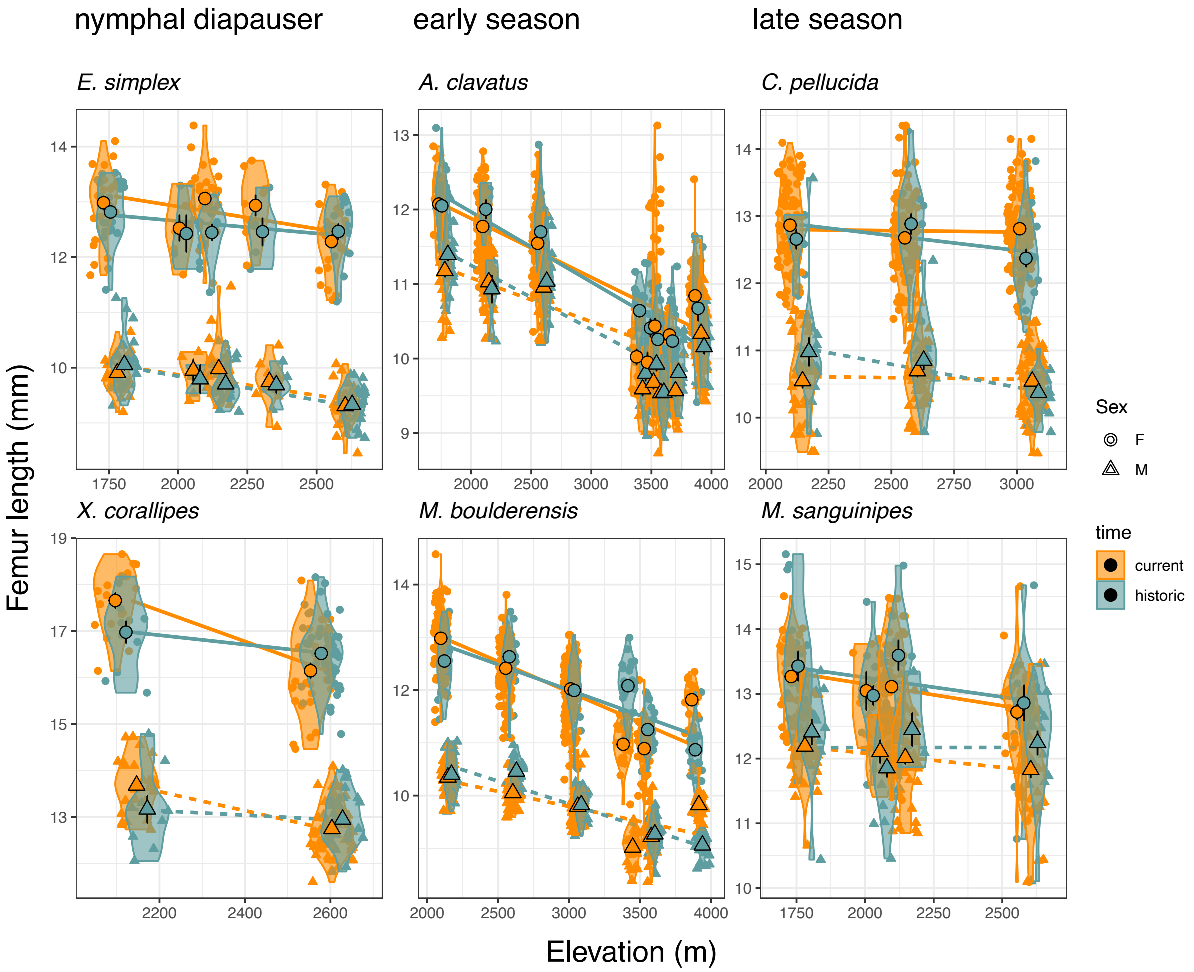

Fig A. Body size (femur length, mm) declines with elevation, females are substantially larger than males, and the body size clines vary with seasonal timing. We depict both violin plots and population means ± SE by sex and elevation. The species are arranged by seasonal timing from the earliest season, nymphal diapausing species to late season species (left to right and top to bottom). Body size changes between the historic and current time period differ based on seasonal timing and elevation. The slope of the relationship with elevation during the historic period (accounting sex as an intercept) generally declines with later seasonal timing (slope +SE= *E. simplex*: -0.40±0.07, *X. corallipes*: -0.45±0.22, *A. clavatus*: -0.48±0.05, *M. boulderensis*: -0.35±0.11, *C. pellucida:* -0.29±0.13, *M. sanguinipes*: -0.11±0.22).

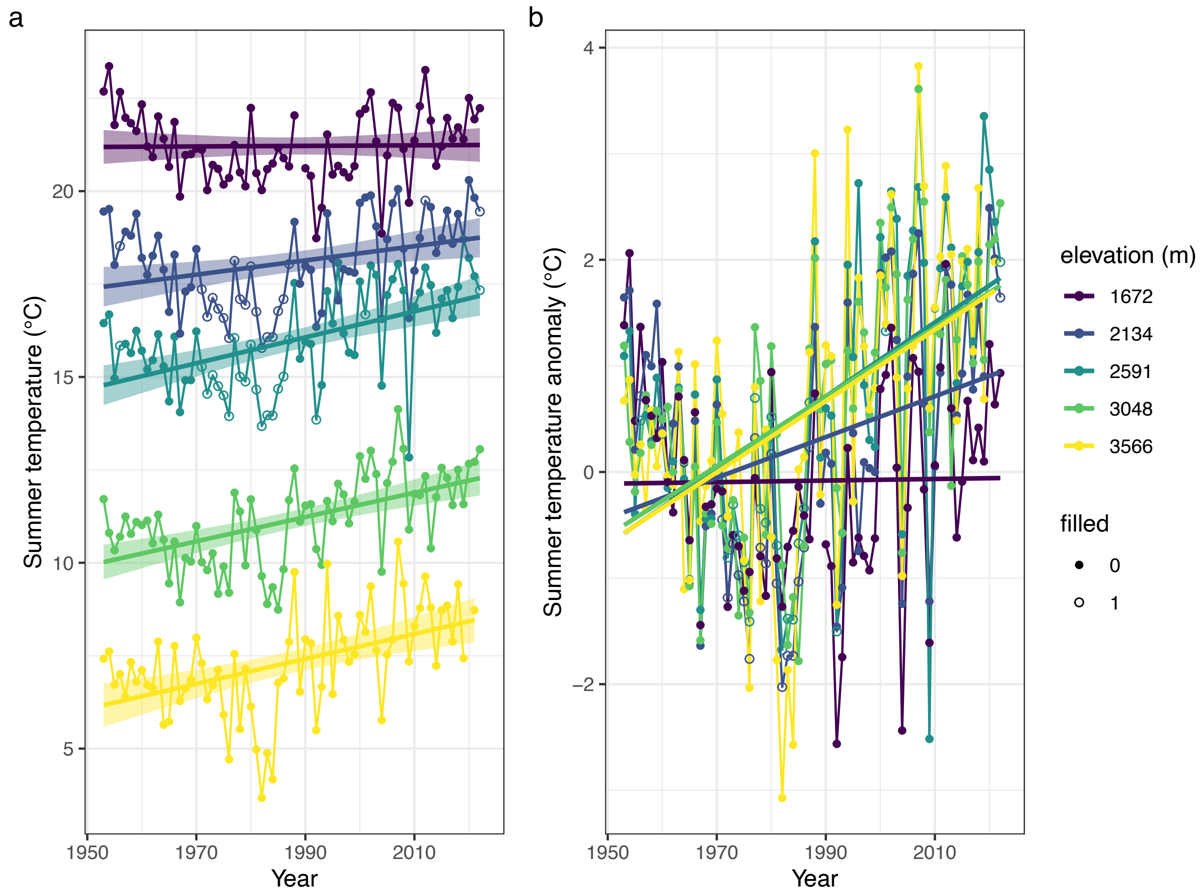

Fig B. Summer temperatures have increased over time. (a) We depict means of daily mean temperature along with linear regression trends ± SE. (b) Depicting the temperatures as anomalies highlights that temperatures have tended to increase more over recent decades at higher elevations (year: $F_{[1,343]}$= 67.01, P<10^{-14}; elevation: $F_{[1,343]}$= 15.46, P=0.001; year * elevation: $F_{[1,343]}$= 14.80, P=0.001). Hollow dots indicate years where data from other sites were used to fill missing data.

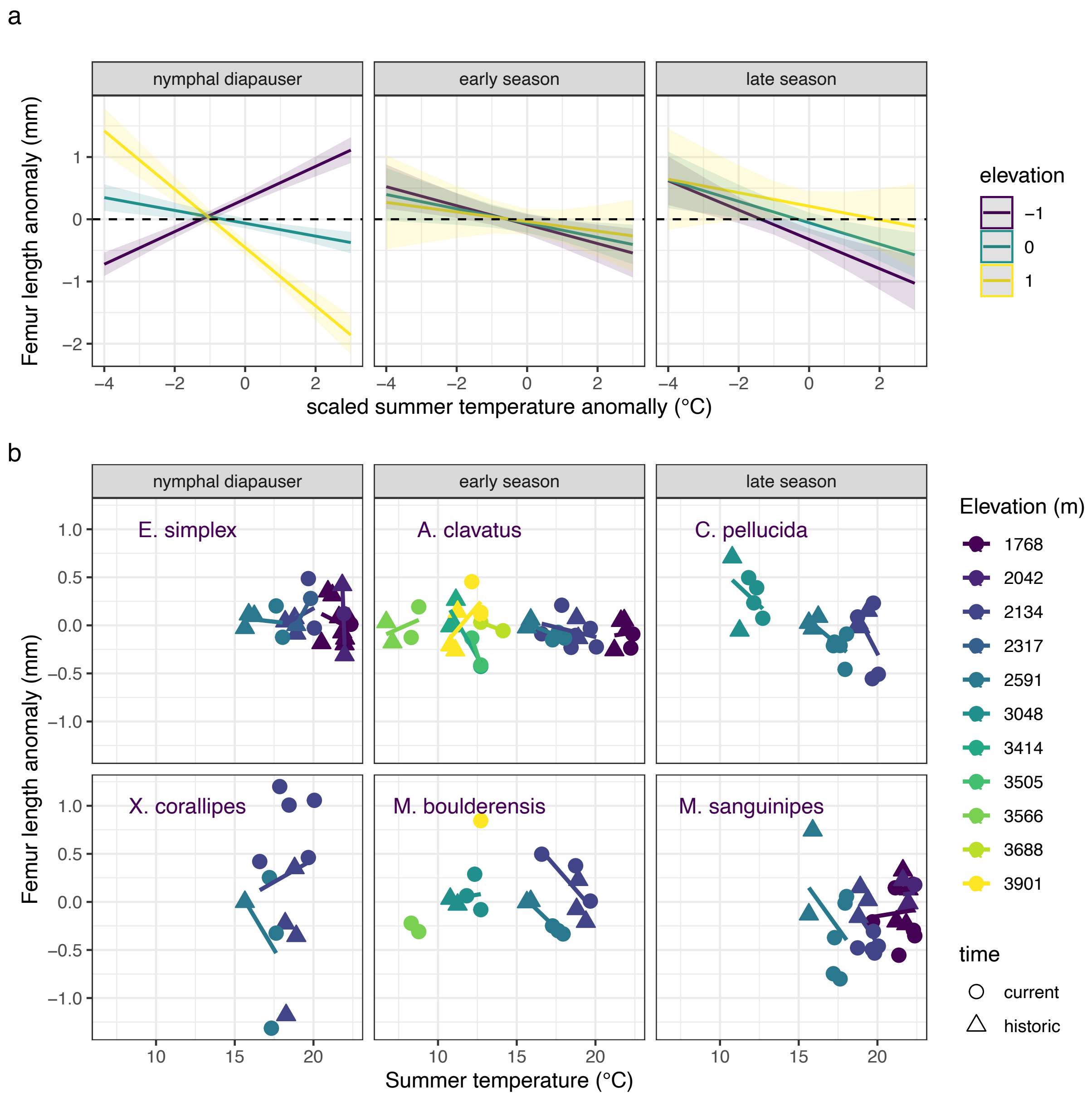

Fig C. Seasonal timing, elevation, and previous summer temperature anomalies interact as determinants of changes in femur length from historic means (accounting for species, elevation, and sex). Nymphal diapausing species have tended to increase size at lower elevations and decrease size at higher elevations in response to warmer spring temperatures. Early seasonal species exhibit mixed responses to spring temperatures. Late season species have tended to decrease size, more strongly at lower elevations, in response to warmer temperatures the previous summer. The trends are depicted as (a) model estimates $\pm$ SE and (b) data means $\pm$ SE per year and site for species arranged by seasonal timing from the earliest season, nymphal diapausing species to late season species (left to right and top to bottom). Data are plotted as a function of spring temperatures rather than anomalies to facilitate visualization.

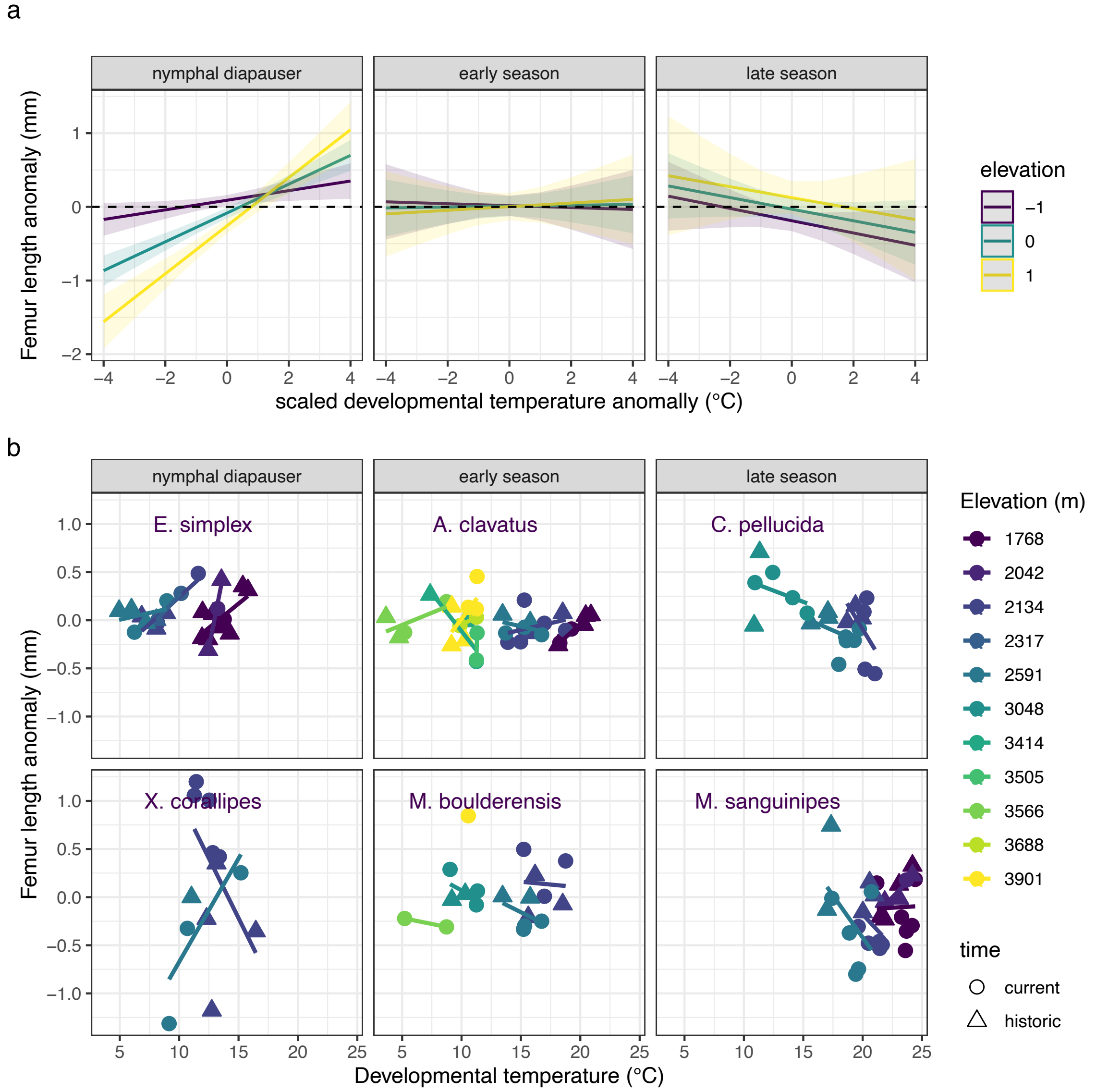

Fig D. Seasonal timing, elevation, and developmental temperature interact to determine changes in femur length in grasshoppers. Nymphal diapausing species have a positive temperature-size response (“hotter is bigger”), with all populations but especially high elevation increasing in size in response to warmer developmental temperatures. Mid-season species do not show consistent responses to developmental temperatures. Late season species have a negative temperature-size response (“hotter is smaller”), tended to decrease size, more strongly at lower elevations, in response to warmer temperatures the previous summer. The trends are depicted as (a) model estimates ± SE and (b) data means ± SE per year and site for species arranged by seasonal timing from the earliest season, nymphal diapausing species to late season species (left to right and top to bottom). Femur length anomalies are mean femur lengths in the modern samples (dates) expressed relative to historic means (dates), accounting for species, elevation and sex. Developmental temperatures are mean temperatures over the 30 days before the collection data of each specimen. Data are plotted as a function of developmental temperatures rather than anomalies to facilitate visualization but were analyzed as anomalies.

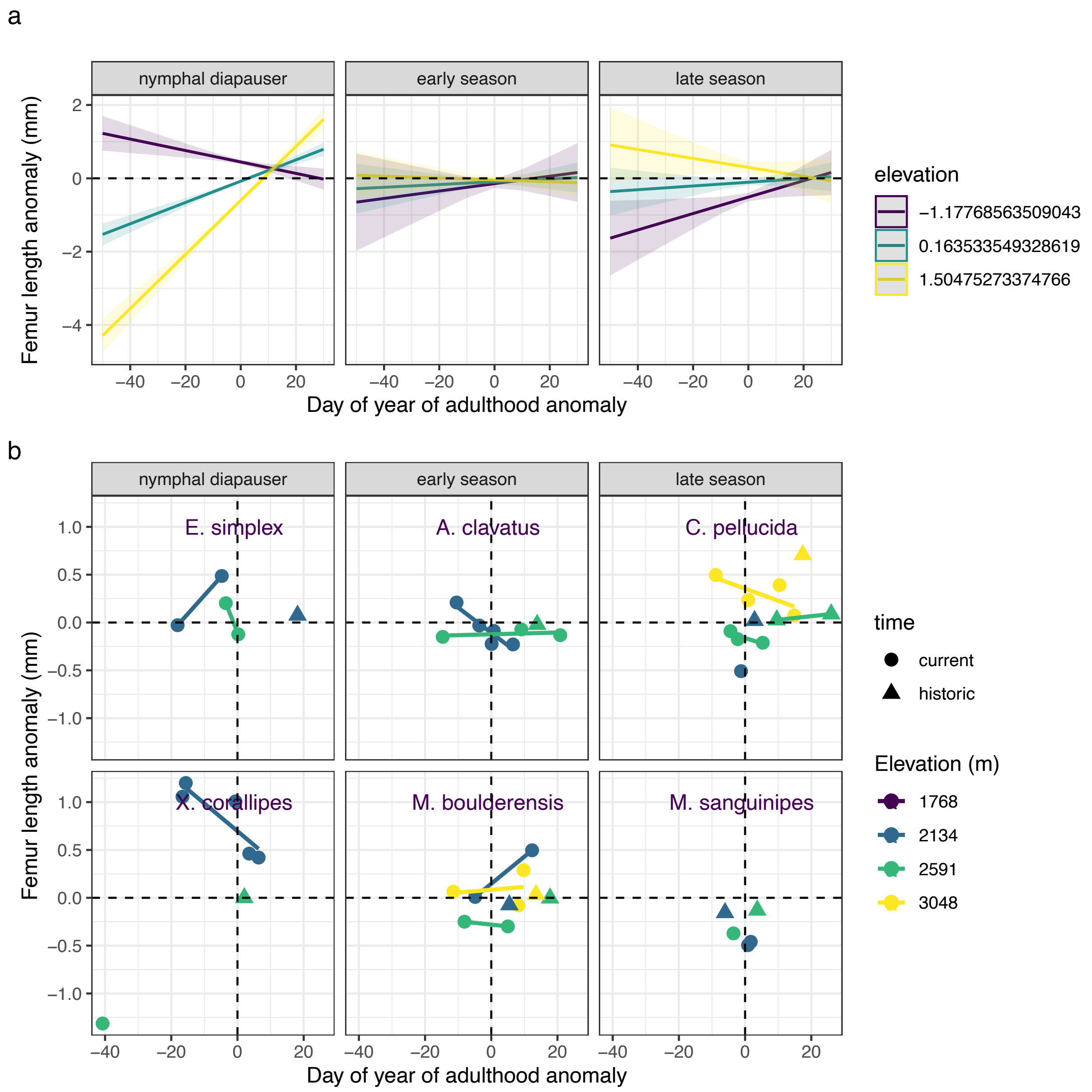

Fig E. Femur length anomalies tend to decrease with later phenology (greater day of year of adulthood anomaly) when phenology is based on field surveys. The trends are depicted as (a) model estimates ± SE and (b) data means ± SE per year and site for species arranged by seasonal timing from the earliest season, nymphal diapausing species to late season species (left to right and top to bottom).

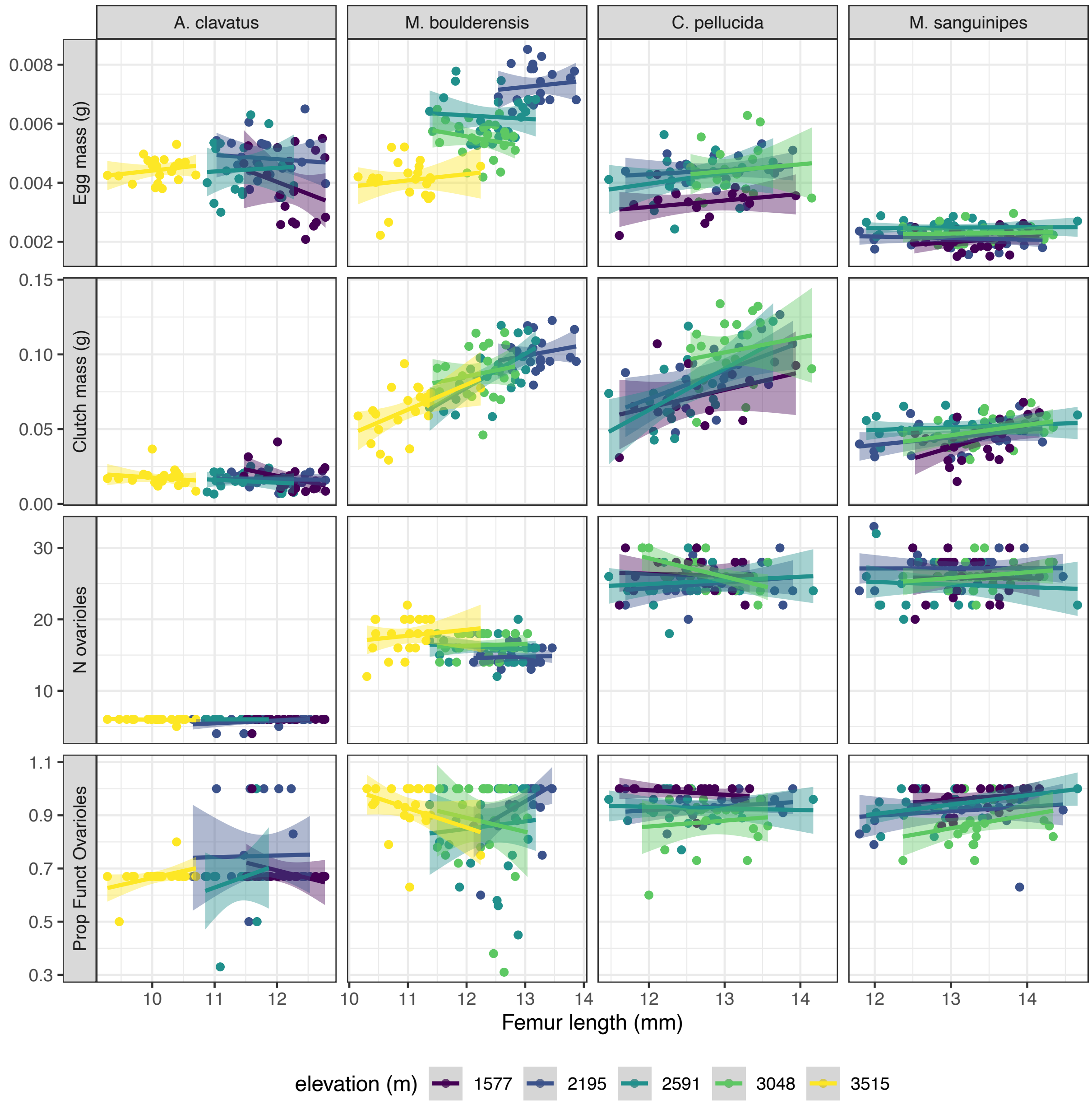

Fig F. Reproductive metrics including (from top to bottom) egg mass, clutch mass, the number of ovarioles, and the proportion of functional ovarioles vary with femur length and elevation (color). Data points correspond to individuals and we depict linear regression trends and standard errors. Data are from Levy and Nufio (35).

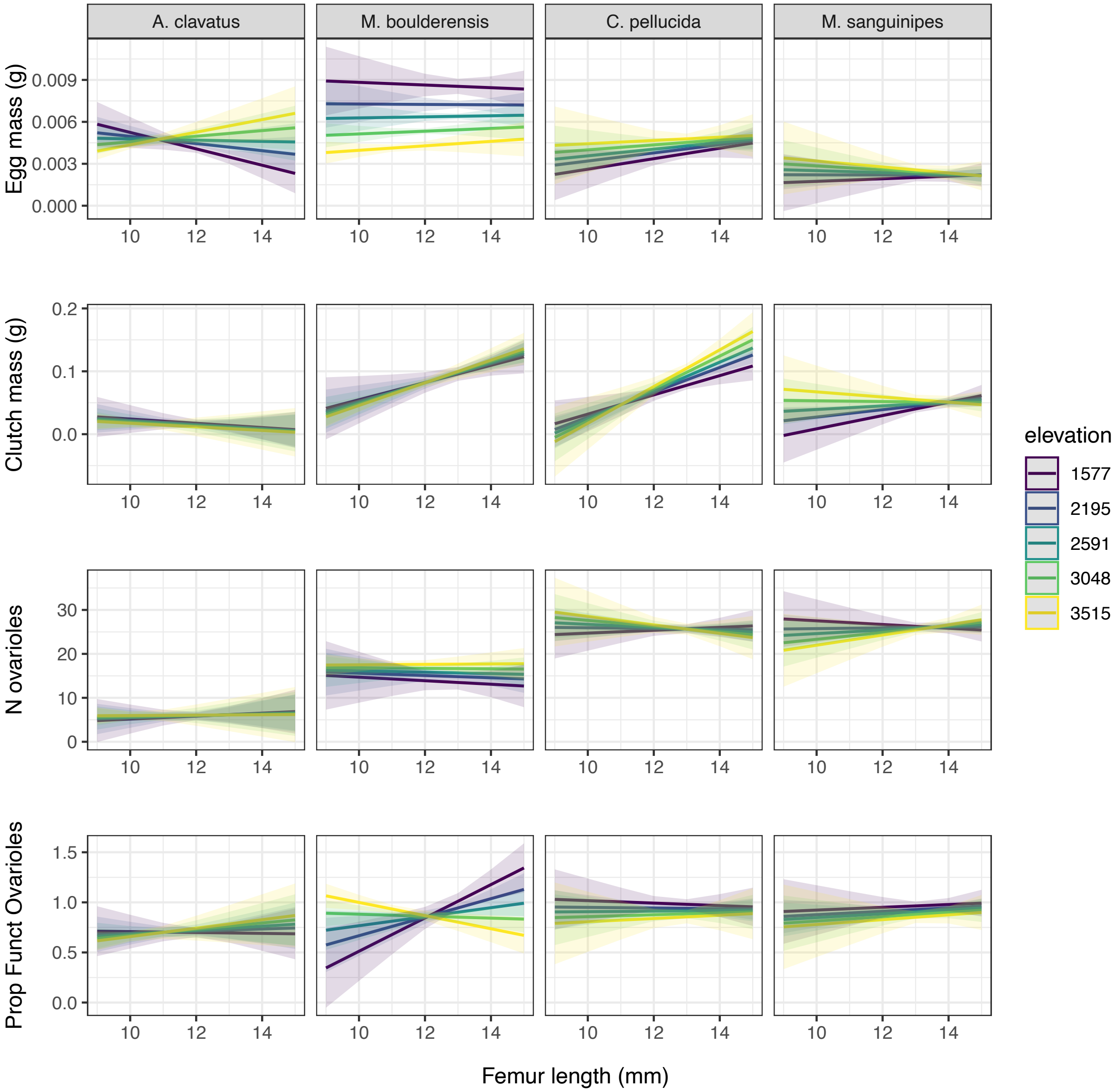

Fig G. Model estimates ± SE indicate that reproductive metrics including (from top to bottom) egg mass, clutch mass, the number of ovarioles, and the proportion of functional ovarioles vary with femur length and elevation (color). Data are from Levy and Nufio (35).
