# Supplementary material for: Insect size responses to climate change vary across elevations according to seasonal timing
Source: PLoS Biol. 2025 Jan 30;23(1):e3002805. doi: 10.1371/journal.pbio.3002805 (PMC11783300; doi:10.1371/journal.pbio.3002805)
Supplement: Attachment — Submitted filename: Buckleyetal_PLoS_response.pdf [file pbio.3002805.s002.pdf]

EDITORIAL COMMENTS:
You'll see that reviewer #1 is very positive about the study, but does (in his enthusiasm) suggest a few more analyses that he says are nice but optional. He suggests that you improve Fig S4 and move it to the main paper, and has a load of textual and presentational suggestions, some of which may involve extra analyses. Reviewer #2 is also positive, and simply wants you to connect it better to the existing literature. Reviewer #3 is positive, but thinks that the motivation is weak, and (like rev #1) suggests promoting Fig S4 to the main paper. S/he has a long list of other textual and presentational requests, and one or two might need a minor analysis.

Thank you for the helpful reviewer suggestions and opportunity to revise the manuscript. We detail our revisions below, which focus on clarifying the motivation and content with the help of a revised conceptual figure. We have also revised several figures as suggested.

REVIEWERS' COMMENTS:

Reviewer #1:
[identifies himself as Wilco C.E.P. Verberk]

General comments:

I very much enjoyed reading this study by César Nufio and co-workers in which they focus on body size responses across elevational clines and multidecadal changes in climate. With 6 species, two time periods and many different elevations there is a lot to unpack and the strength of this study lies in the clarity with which things are presented and explained as well as the unique dataset. I made some suggestions below, which will hopefully help the researchers to further improve or clarify their manuscript. In my enthusiasm, I also suggested some additional analyses; I realise the supplementary materials is already packed with analysis and the author will be in the best position to decide if those analysis are useful or only lead to rabbit holes that are either already explored or will detract from the main message of this manuscript.
Signed, Wilco Verberk

Detailed comments:

Line 51: this statement about small-bodied organisms likely pertains especially to terrestrial small-bodied organisms. Note that for aquatic animals there are patterns between body size and heat tolerance limits, but these are generally negative, i.e. small bodies organisms have higher peak body temperatures.

We now specify terrestrial.

Line 54-59: Note also figure 5 in ref 7 where a link between voltinism and size responses is made. Voltinisms may be particularly important in terrestrial environments where end-of-season-constraints are harsh (e.g. drought and frost), which are attenuated at least somewhat in an aquatic environment.

Thanks for this point. The wording of “when seasons are short” should hopefully account for this difference between terrestrial and aquatic systems.

Line 69: In this light, it may be better to replace 'insects' in the title by 'grasshoppers'?

We prefer to keep insect since similar mechanisms are likely to mediate variation in responses across elevations and species with different seasonal timing across many insects, but we can change to grasshoppers if preferred by the editors.

Line 81: I found fig S4 potentially worthwhile to include as a conceptual figure in the introduction, but somewhat confusing in its present form: in the left panel, I see all different hypotheses, but some very specific ones were made (as far as I could tell, the intro suggests the ascending line of low elevation shrinkage and high elevation enlarging). The y-axis is basically a temperature axis as it compares historic and current sizes and the main difference is temperature. In that sense the right panel should not really have a thermal cline as the comparison is already between historic and present which incorporates a temperature comparison. So I don't follow what the different lines do. Note that this panel would make a lot more sense if it just had size on the y-axis rather than anomaly. Also, since the paper to this point has been mostly about temperature (not altitude or historical warming), it may be clearer to lead with that panel.

Thank you for these suggestions. We have simplified the figure to focus on temperature responses with a second axis for elevation clines. We now focus on temperature-size rule (TSR) and reverse TSR responses and how thermal opportunity and stress can drive divergences from the expectations. We have moved the figure to the main text as also suggested by another reviewer.

Line 90: Although the introduction lays out the framework and an overarching hypothesis, the data are quite complex and breaking it down into subquestions each addressed by a different analysis and paragraph could help. For example, this paragraph asks about the elevational cline and whether this is different depending on a species' seasonal timing. This is important to establish but does not yet deal with the comparison between historical or current data. In the next paragraph this follow up question could then be asked. Note that from Table 1, there does not seem to be a strong effect of Time, which agrees with me eyeballing figure 2.

We have now broken down the results into sub-sections addressing clinal patterns and their shifts, temperature responses, and links with phenology.

Line 92: I can see why the size cline is referred to as a hotter is better pattern, but the high elevation populations presumably have less time and the greater time constraints likely select for faster development or a greater thermal sensitivity of development rates (as I see has been observed for M. boulderensis in line 214). So this pattern could have also arisen by effects other than temperature. Note that with all the temperature data, it is possible to convert the elevational points into a temperature cline and then see if decreasing body size with increasing elevation can be explained by increasing temperatures at low elevation. By including interactions between this temperature gradient and both altitude and time, one could even test to what extent temperature size-responses are modulated by either altitude or evolution.

Note that we are referring to “hotter is bigger”, rather than “hotter is better”, which we uncover from controlled lab experiments in addition to inferring from field observations. Since ectotherm growth and development rates are temperature-dependent, it is difficult to isolate determinants of growth and development from temperature. The suggested analysis is related to Fig 1 in Nufio and Buckley (2019, Ecosphere, **https://doi.org/10.1002/ecs2.2866**). Our past work has

shown that there is indeed selection for faster development and greater thermal sensitivity, so we omit related analyses here. We have attempted to strengthen connections to this past work in the discussion.

Line 92: To drive home the point that the slopes of the elevational relationships are shallower with later seasonal timing, I would plot all panels with the same range for the x-axis and y-axis and express the slopes as a percentage of 'shrinkage' to account for size differences across species.

Thank you for this suggestion. We now plot femur length as a proportional difference from the species' mean size across the historic period. This allows for the same y-axis range. We have not used the same x-axis range because it makes it difficult to visualize the data.

Line 99-105: This is basically the stats to underpin the previous paragraph? So maybe just incorporate there? Also, were two analyses performed (one on historic data, one on current data? Why not include historic and current as a factor to provide different intercepts?

We moved the first mention of the elevational clines down to after we describe the statistical analyses. We initially conducted the analyses separately by time point to explore how the elevational clines within each time period differed by sex and according to seasonal timing. Analyzing all data together and adding time as a factor would have led to the models becoming too complex and impossible to interpret. Our subsequent analysis explores the effects of time period using body size anomalies, removing the main effects of species, elevation, and sex and looking only at changes in body size between time periods.

Line 102: How do these F-values relate to those quoted in table 1?

Table 1 describes changes in body size anomalies through time whereas the indicated F values describe historic body size clines. We now clarify this in the manuscript by explaining the second set of models more explicitly.

Line 104: Given the significant 3-way interaction that includes sex, I wonder why sex does not feature in much of the rest of the manuscript? Many of the reasoning (e.g. capitalizing on permissive conditions for growth) will be more or less important depending on the sex of the animal. Note that this interaction also seems to run against line 339

As briefly mentioned in the methods, size responses to warming were qualitatively similar across sexes. We initially included sex in the models and figures, but we found that including sex decreased intepretability without altering the findings. We controlled for the size differences associated with sex by calculating anomalies.

Line 109: This refers to the time:elev:SpTiming interaction in table 1 ($P=0.02$)? Not that this effect is not included in the best model and not significant in a model that also includes temperature ($P = 0.76$).

Yes, that is the reference. One of our points is that changes in temperature across years and time periods can account for differences in size across elevations and time. We have clarified our reasoning in the text.

Line 113: I guess figure 3 is based on the first model in Table 1? I would suggest to express the

femur length anomaly as a percentage to prevent larger species to have greater anomalies just because they are larger, rather than being more plastic in size.

Yes, figure 3 corresponds to the first model in Table 1. We have changed figure 3 to depict femur length differential as a proportion.

Line 118: In figure 4, it seems that femur anomalies are all over the place for nymphal diapausers, until I realised that both species are both inhabiting the lower altitudus. Instead of showing the full range of the (scaled) elevation in these plots, maybe only show model preductions for the upper and lower elevational range for the species involved (I very much appreciate the constancy of colors to denote elevation)?

We appreciate this suggestion, but concluded that it would be confusing for the model estimates to vary in elevation between panels. We labeled the high-elevation model estimate for nymphal diapausers, which we now point out in the legend: "Since we did not sample nymphal diapausers at high elevations, we have labeled the high elevation model estimate".

Line 145 So, if you were to plot absolute doy vs absolute size for each of the two time periods one would see a negative relationship (i.e. animals taking longer to develop will reach a smaller size at adulthood)? This is strange in the context of the TSR where one frequently assumes there is a case of temperature shifting the trade-off towards maturing early at a small size rather than later at a large size.

Yes, it is strange and puzzling, but the effect seems to correspond to relevant biology both in the field and lab. We hold the effect results from the non TSR effects depicted in the revised Fig. 1 (thermal opportunity at low temperatures; thermal stress at high elevations).

Line 175 In this sense it could be interesting to see if the size-doy relationship for these late season grasshoppers is different in anomalously hot years or not.

We agree this would be an interesting analysis. However, our phenological data is not sufficiently resolved for a meaningful analysis. Our phenology data either rely on field population estimates or specimen collection dates, which do not sufficiently resolve within season phenology given that adults can live for quite a white.

Line 179: maybe I missed this, but where is this in the results? I think this is a bit tangential as the paper focusses on seasonal timing of species as an important driver for size anomalies. Also, since nymphal diapausing species (with steep slopes) are low elevation, I wonder to what extent one can link body size changes unambiguously to elevation. If you need to cut something from the ms, I think this paragraph is a good candidate unless I missed something.

We have added a reference to Figure 4. Yes, the finding is confounded with nymphal diapausing species primarily occurring at low elevations.

Line 190 Something which is further corroborated by the strong effect of temperature in the 4 weeks preceeding adult measurements?

We now indicate that "Strong temperature responses are also evident when examining developmental temperatures (Table S2)" (Line 197).

Line 216 Allow me to highlight a study in butterflies where growth and development were also modulated by an interaction between temperature and photoperiod, although the size response was much buffered as growth and development were similarly affected (https://urldefense.com/v3/__https://doi.org/10.1016/j.cris.2022.100034__;!!K-Hz7m0Vt54!kbWEeBGhuqOb4vM3lez_v2inByMVygg-GILpjA56vn641YXUnsPUzMWKkCCbzqDiRCnyPJncG9tRFB5M$)

Thanks for the reference suggestion, which we added to the beginning of the paragraph.

Line 220: Instead of referring to fig 5, this could refer to (a modified, clearer version of) Fig S4?

We now refer to the conceptual figure as suggested, which is now Fig 1.

Line 230: A problem with multi-species comparison, is that species will differ in more respects than that which one is interested at. A possible solution could be here to focus on differences between males and females. Do males and females differ in their trade-off between growth (ie size) and development (i.e. time or doy) and how is this influenced by cues of temperature and photoperiod. For males, it is beneficial to not just grow as large as possible, filling up the whole period of permissible growth season, but rather to also be sufficiently early to maximise mating opportunities. I wonder if the 3-way interaction between elevation, sex and seasonal timing (line 104) can be leveraged to study how prioritizing size and age is a function of temperature and time available (i.e. photoperiod).

We appreciate this suggestion, but as discussed above responses are similar across sexes. Some differential responses between males and females in controlled lab experiments are consistent with trade-offs between growth and development (e.g., Smith et al. 2021), but we don't feel we have the control in this dataset for such analyses.

Reviewer #2:

In the MS "Insect size responses to climate changes vary across elevations according to seasonal timing" by Nufio et al. the authors explore the effect of warming on insect development.

The study reports an increase of growth season temperatures along an elevational gradient using temperature records for the last 60 y. The study compares length of femurs as an estimate of size for six species of grasshoppers. The life histories of these species show different seasonal timing that interact with changing temperatures along the elevational gradient. The study shows that size responses are complex and depends on the timing at which species start their development. Early species show an increase of size, but late species show a decrease of size.

This study takes advantage of a unique historical dataset to determine the complex effects of increasing temperatures on insect morphology, and maybe population dynamics. My only concern is that this study needs to connect the results with the broader context, and general theory.

My suggestion to the authors is to include in the introduction some of the theoretical expectations of ecogeographic rules.

For example, the authors should discuss how their findings connect to previously proposed

patterns such as 'James' rule', which focuses on the relative effect of temperature and body size:

James, F. C. (1970). Geographic size variation in birds and its relationship to climate. Ecology, 51, 365-390. https://urldefense.com/v3/__https://doi.org/10.2307/1935374__;!!K-Hz7m0Vt54!kbWEeBGhuqOb4vM3lez_v2inByMVygg-GILpjA56vn641YXUnsPUzMWKkCCbzqDiRCnyPJncG7nzeJaJ$

Or the 'temperature-size rule', which states that body size tends to decrease with increasing temperature.

Atkinson, D. (1994). Temperature and organism size: A biological law for ectotherms? Advances in Ecological Research, 25, 1-58.

The manuscript will also benefit to discuss if ectotherms should be considered under Bergmann's rule.

Blackburn, T. M., Gaston, K. J., & Loder, N. (1999). Geographic gradients in body size: A clarification of Bergmann's rule. Diversity and Distributions, 5, 165-174.

Daufresne, M., Lengfellner, K., & Sommer, U. (2009). Global warming benefits the small in aquatic ecosystems. Proceedings of the National Academy of Sciences of the United States of America, 106, 12788-12793.

We now introduce James' Rule and Bergmann's Rule in addition to the temperature size rule: "Organisms tend to be smaller in warmer geographic locations (e.g., low latitude and elevation) both within (James' Rule) and across species (Bergmann's Rule). Both rules encompass evolutionary drivers. Both rules were originally formulated for endotherms and have mixed support for ectotherms (9)".

In the discussion, the authors may include the potential effects of oxygen partial pressure and air density.

Dillon, M. E., Frazier, M. R., & Dudley, R. (2006). Into thin air: Physiology and evolution of alpine insects. Integrative and Comparative Biology,

Callier, V., & Nijhout, H. F. (2011). Control of body size by oxygen supply reveals size-dependent and size-independent mechanisms of molting and metamorphosis. Proceedings of the National Academy of Sciences of the United States of America, 108, 14664-14669

Thank you for this suggestion, but we do not think our sites are high enough to warrant a discussion of oxygen partial pressure or air density.

Also, the authors may discuss the potential phylogenetic effects on their results.

The species are closely related and we were unable to readily resolve the phylogeny based on genetic sequences (Buckley et al. 2021 JAE). We thus do not expect phylogenetic clustering to influence our conclusions, so have not added a discussion of phylogeny.

After these minor changes, I think the study is an excellent contribution to understand how ectotherm size is affected by increasing temperatures.

Thank you for the appreciation.

Reviewer #3:

Paper Summary
In this paper, the authors used natural history collections and modern field observations to investigate how the body sizes of grasshoppers have changed over 60 years of recent climate change. The study system was an assemblage of six grasshopper species living along a 2000-m elevation gradient in the Rocky Mountains. The authors compared the body sizes of grasshoppers collected from 1958-1960 to the body sizes of modern samples collected from 2006-2015. By fitting linear mixed-effect models, the authors analyzed how species traits interact with climate, time, and elevation to influence the body size of grasshoppers. These analyses enabled the authors to evaluate competing hypotheses for how temperature affects body size ("hotter is smaller" vs "hotter is bigger") while documenting changes in body size in response to climate change. The authors found that body size responses varied among species and life histories. As the climate has warmed, early-season species, especially those at low elevations, tended to develop faster and grow larger. In contrast, warming reduced the body sizes of grasshoppers that develop later in the season and live at higher elevations, underscoring the idiosyncratic nature of climate change responses and the complex relationships between temperature and body size.

Strengths
The greatest strength of this paper is its successful integration of multiple approaches. The authors combined historical collections, modern sampling, and quantitative analyses to document climate change responses and test eco-physiological hypotheses. These datasets and analyses are thoroughly described and scientifically sound, and the authors have produced clear figures that effectively communicate the results.

Weaknesses
A weakness of this paper is the stated motivation; the authors assert that declining body size has been proposed as a universal response to warming. In my view, no reasonable biologist would predict a universal response, and relatively recent investigations of this topic have confirmed that there is variation in how body size responds to warming (e.g., Diamond et al. 2010; Kingsolver and Huey 2008; Ashton and Feldman 2003). I believe that the paper would be strengthened by a focus on competing hypotheses for body size responses, rather than evaluating evidence for a "universal response". I especially appreciate the ideas behind the author's conceptual S4 Figure, which outlines two competing hypotheses. Although I am confused by some of the lines in the left panel, I recommend elevating this figure to the main text and framing the study as an investigation of those competing hypotheses.

We continue to feel that discussing a universal response to warming is justified given the cited papers, but we have minimized the discussion of universal responses to focus on explaining observed variation in responses. We note that the suggested papers mostly address responses to long term thermal clines, which have been found to be more variable than climate change responses. We have simplified the conceptual figure to more closely align with the text and have moved it to the main text as suggested.

Paper Assessment
Overall, this paper successfully integrates approaches to investigate the impacts of climate change on grasshoppers. It is a well-written paper and well-conceived project that will be of

interest to a broad group of ecologists, physiologists, and entomologists. I hope that my comments can reinforce the clarity and impact of the paper.

Specific comments

Abstract

Overall: This abstract would be more effective if it included more details about the study and results. My main recommendation is to state how exactly body size and seasonal timing have changed for these grasshoppers on average (e.g., % change in femur length). Similarly, consider specifying more about the study design, including the time periods, number of species, elevation range, etc.

We have added additional study details to the abstract: “Leveraging long-term data for six montane grasshopper species spanning 1768 to 3901m, we detect size shifts since ~1960 that depend on elevation and species’ seasonal timing”. We do not report average changes in femur length because we feel doing so would undermine our point that body size changes have been variable across elevations and life histories.

L18-20: I am wondering how many biologists would argue today that body size declines are a "universal response to warming". Huey and Kingsolver 2008 (cited by the authors) found that warming reduced the body size of only 80% of the studied organisms, demonstrating 16 years ago that this response is not universal. Consider revising this opening sentence to identify a clearer knowledge gap and clarify the prevalence of body size declines among diverse taxa.

As discussed above, variation in response to long term temperature clines is more recognized than in response to warming. The similarity of thermal responses at the two time scales is an interesting (and still fairly open) question. We have revised the opening to remove the reference to universal responses: “Body size declines are a common response to warming via both plasticity and evolution, but variable size responses have been observed for terrestrial ectotherms”.

L21-22: It seems like "universal size responses" is being used as a stand-in for predictions from one hypothesis (i.e., temperature-size rule), which is known to not be universal. Is there a more nuanced way to present the evidence of body size responses?

Increased sizes with warming do not necessarily stem from the TSR (particularly since size increases are better studied in endotherms), but we have rephrased as: “We investigate how temperature dependent development and growth rates in ectothermic organisms induce variation in size responses”.

L23-24: Starting at "Size shifts have been…" I think there is a misplaced comma in this sentence, and the sentence is hard to follow. Consider splitting into two sentence or otherwise revising for clarity.

We revised as follows: Size shifts have been concentrated at low elevations, with early-emerging species (those that overwinter as juveniles) increasing in size, while late-season species are becoming smaller.

L27: Please clarify whether this hypothesis was formulated a priori or after the results were known. If the former, I recommend introducing this hypothesis earlier in the abstract. If the latter,

I recommend making it clear that the authors used the results to formulate the hypothesis.

The hypothesis was formulated a priori and is included in our grant proposal from 2020. However, in response to further feedback below regarding partial support of our hypotheses, we have decided to remove reference to the hypothesis in the abstract and explain it more fully in the introduction.

L37-38: Citations 1 and 2 seem to also refute the idea of universal size responses to warming. There is reason to believe that many organisms will shrink, but few biologists, if any, would have argued that this response would be universal… Perhaps this first sentence of the introduction can be deleted to make way for an argument from first principles as to why body size declines (or increases) might be expected.

We feel that it is important to acknowledge the common assumption of body size declines, which the references address. We have rephrased to "Declining body size is a commonly anticipated response to anthropogenic climate warming" (L35).

L46: I believe there should be a dash between temperature and size (temperature-size rule).

Change made.

L46-48: Consider introducing Bergmann's Rule, which seems relevant to the TSR and the relative support for endotherms vs ectotherms.

We now introduce James' Rule and Bergmann's Rule in addition to the temperature size rule: "Organisms tend to be smaller in warmer geographic locations (e.g., low latitude and elevation) both within (James' Rule) and across species (Bergmann's Rule). Both rules encompass evolutionary drivers. Both rules were originally formulated for endotherms and have mixed support for ectotherms (9)".

L68-74: I enjoyed reading this clear description of why grasshoppers tend to reverse the TSR.

Thank you.

L78-81: This section presents a detailed hypothesis that is partly refuted and partly supported by the subsequent analyses, yet the abstract gives the impression that the authors' findings were entirely "consistent with our hypothesis". Consider fully detailing this hypothesis in the abstract.

As discussed above, we feel the hypothesis can best be detailed in the introduction, so we have removed reference to the hypothesis in the abstract.

Results

L93-98: This information about the species and their seasonal timing might make more sense in the Methods to illustrate how the authors deliberately selected the species to control for other sources of variation (e.g., in voltinism).

Given that the methods appear at the end, we feel it is important to introduce the species here to aid interpretation.

L97: The authors call A. clavatus and M. boulderensis mid-season species here, but elsewhere, it seems like "early-season" is used.

Thanks for catching this. Change made.

L104: The methods (L339) state that sex was omitted from the models, yet sex differences are graphed (Fig. 2) and reported. Please clarify in the methods.

The sex differences were included in plots of size clines (Fig. 2) but were omitted in analyses of climate change responses as discussed above. We now specify: "preliminary models indicated that size responses were similar across sexes, so we omitted sex from our models of climate change responses" (L338). Sex differences in size were accounted for by calculating anomalies.

L109-112: Add a semi colon in place of the comma on L112 to fix the run-on sentence

Done.

L111: In Figure 3A, I am surprised that the model estimates such weak effects of elevation on the body size of mid-season species (M. boulderensis and A. clavatus) because there seem to be strong body size clines for these species in Figure 2. What causes this discrepancy?

The species are relatively small, which contributes to weak effects of elevation despite strong elevation clines.

L134-136: Consider adding more detail to explain biologically what this 4-way interaction implies. This might make it easier for readers to see how this is evidence for evolutionary change. For example, "The 4-way interaction suggests that grasshoppers at different elevations and with different life histories are responding differently to temperature now than they did historically" or something to that effect.

We adopted the suggested wording: A 4-way interaction suggests that grasshoppers at different elevations and with different seasonal timing are responding differently to temperature now than they did historically, indicating the potential for evolutionary responses between time periods (L140).

L136: Is "Extended Data Table S1" different from "Table S1"?

No. Fixed.

L145-150: The objective(s) of these analyses were not clear to me.

We have added a subheading "body size and phenology" to clarify the objectives. Given that phenological shifts are a primary documented response to warming, we feel it is important to discuss the link between body size and phenology and detailed in the discussion.

Figure 2: These graphs are not intuitive because they all have different scales on the x and y axes. Consider standardizing the scales wherever possible, although I know that will create other data visualization problems.

As suggested by reviewer 1, we have changed the y-axis to proportion to allow for a common axis.

Figure 3A: The yellow shading is quite hard to see. Consider using another color with more contrast. Also, double check the paper for use of nymphal diapauser vs early season vs mid season. The authors introduce "mid-season" on L97, but it is not used elsewhere. I happen to like the term mid-season to more easily distinguish from nymphal diapausers.

We have revised the colors in the model output to align with the current and historic colors below.

We have changed the reference to “mid-season”. We had previously tried referring to early and mid season species rather than nymphal diapauser and early season species. We ultimately used the clunkier nymphal diapauser, early season, late season terminology to align with some previous publications on the system that omitted nymphal diapausers.

Discussion

Overall: Discussion would be strengthened by adding references to tables and figures to remind readers of where to find evidence for various claims.

We have added references to tables and figures as suggested.

L159-160: The authors' focus on universal responses is misplaced. A universal response would be evidenced by every species always responding in the same direction, but no reasonable biologist would have predicted a universal response in body size. I think the paper and its conclusions would be stronger if the authors framed their study and conclusions as providing evidence to distinguish between competing hypotheses, rather than as providing evidence for or against a "universal response".

We removed most references to universal responses. The sentence now reads: “Using these highly standardized natural history collections, we found that life history and environmental differences can account for variable relationships between temperature and body size” (L167).

L162-164: What statistical evidence supports the claim that "seasonal timing was the most important predictor of response to climate change"? I am specifically wondering how we know that seasonal timing is a more important predictor than say species. Also, this statement that "changes were explained by divergent phenotypic plasticity in species with contrasting life histories" seems too strongly worded. Given the correlative nature of the analyses, how confident are the authors that these changes are explained by phenotypic plasticity? And how confident are the authors that contrasting life histories is the mediating factor? It seems to me that this is best framed as a newly formed hypothesis, not a final conclusion.

We agree that the statement regarding “the most important predictor” was not statistically justified. We intended to indicate that seasonal timing can help clarify the origin of variability in size responses. We now specify: “we found that size responses to warming across elevation were relatively consistent among species with similar seasonal timing, potentially due to responses to thermal opportunity and stress (Fig 1)” (L169).

L168: Note that life history differences are just one explanation for the different responses to climate warming. There are many other traits that might be better predictors but were not included here.

Additional differences between species are discussed in the methods. Consistent responses among species with different seasonal timing, which are consistent with past laboratory experiments investigating mechanisms, suggest the importance of seasonal timing.

L170: I recommend referencing Figure 3 at the end of this sentence.

Done

L186-191: The authors also found strong support for a 4-way interaction with temperature, time period, seasonal timing, and elevation, supporting the idea that the body size response to temperature is different today than it was previously. How does this evidence factor into their conclusions about evolutionary change vs phenotypic plasticity?

We now discuss the 4-way interaction: "We do detect a persistent 4-way interaction between time period, previous summer temperatures, elevation, and seasonal timing that could indicate evolutionary responses (Table S1)" (L198).

L205-208: What is meant by "completion" here? Is it when the nymphs arrest development ahead of overwintering? Or is it when nymphs completely develop to adulthood following overwintering?

We now refer to the "resumption of development".

L220: I do not understand why the authors are referencing Figure 5 here. Perhaps a typo and Figure S4 would be better?

We now refer to the conceptual figure, which is now figure 1.

L243: I do not see how the effect of phenology is a universal response when there are positive and negative slopes shown in Fig S8, panel A.

We have overhauled this section to avoid referring to phenology as a "universal response".

Methods

L284: How were the focal species selected? I assume that the historical collections and modern sites have more species than those studied here?

We now specify that we "focused on species that were well represented in the collection across time periods and elevations (L280).

L291: Replace "spanning" with "at".

Done.

L333-334: I would appreciate more justification for why the authors included species as a random effect rather than a fixed. I suspect that it is related to the fact that the authors are primarily aiming to estimate the effects of life history, not species, on femur length, but it would be good to clarify. Similarly, I am curious whether model performance is higher when species is included as a fixed effect instead of life history.

We now specify that “species was included as a random effect since we are focused on life history differences between species, but models that included species as a fixed effect performed similarly” (L332). Indeed, model performance is similar when species is included as a fixed effect instead of life history.
