# Supplementary material for: Insect size responses to climate change vary across elevations according to seasonal timing
Source: PLoS Biol. 2025 Jan 30;23(1):e3002805. doi: 10.1371/journal.pbio.3002805 (PMC11783300; doi:10.1371/journal.pbio.3002805)
Supplement: Attachment — Submitted filename: PLOS_response.pdf [file pbio.3002805.s003.pdf]

Dear Roli,

Thank you for the review of our manuscript. We have addressed the remaining issues as detailed below.

Lauren Buckley and of behalf of co-authors

Thank you for your patience while we considered your revised manuscript "Insect size responses to climate changes vary across elevations according to seasonal timing" for publication as a Research Article at PLOS Biology. This revised version of your manuscript has been evaluated by the PLOS Biology editors and the Academic Editor.

Based on our Academic Editor's assessment of your revision, we are likely to accept this manuscript for publication, provided you satisfactorily address the following data and other policy-related requests.

a) Please can you modify your Title very slightly by removing the "s" from "changes"? We appreciate that you did assess multiple aspects of climate change, but this will be readily apparent in your Abstract, and the presence of the phrase "climate change" in the Title will, we think, enhance discoverability.

Thank you for this suggestion, which we implemented.

b) You rightly say that no ethical approval was needed, but did you require a field research licence for the sample collection? Please include this information if applicable.

A permit is generally not needed to collect the grasshopper species, but we have had approval of the collection since it was part of field research that did require permitting. We now specify in the methods:

"Approval for resurvey field research and specimen collection was provided by the US Forest Service, City of Boulder Open Space and Mountain Parks Department, and University of Colorado Mountain Research Station."

c) Please address my Data Policy requests below; specifically, we need you to supply the numerical values underlying Figs 2AB, 3, 4AB, 5AB, 6AB, S4, S5AB, S6AB, S7AB, S8AB, S9, S10, either as a supplementary data file or as a permanent DOI'd deposition. I note that you already have an associated GitHub deposition, containing a nicely organised set of code that I suspect, along with the data in Dryad, might enable readers to reconstruct the Figs. Please can you confirm this?

The code has been available in Dryad, but we have now revised the code and published the repository. Data and code are also available at https://github.com/lbuckley/HopperBodysize.

d) Because Github depositions can be readily changed or deleted, please make a permanent DOI'd copy (e.g. in Zenodo) and provide this URL (see below). An potentially neater alternative would be to dump a copy of the Github code into your Dryad deposition, so that everything's in the same place...

e) Please cite the location of the data clearly in all relevant main and supplementary Figure legends, e.g. "The data underlying this Figure can be found in S1 Data" or "The data underlying this Figure can be found in https://urldefense.com/v3/__https://zenodo.org/records/XXXXXXXX__;!!K-Hz7m0Vt54!mgkdhC8HOjugc9vRlqrWjvuWsEecUh5jj235mqcEOO0Wy24OaPbcqTZSUc5Gn2BuLCGNmCrhOXABfVQe$ " or "The data and code needed to generate this Figure can be found in https://urldefense.com/v3/__https://doi.org/10.5061/dryad.wwpzgmst6__;!!K-Hz7m0Vt54!mgkdhC8HOjugc9vRlqrWjvuWsEecUh5jj235mqcEOO0Wy24OaPbcqTZSUc5Gn2BuLCGNmCrhOTCf9jK9$ "

Each figure now specifies: “The data and code needed to generate this Figure can be found in Dryad (https://doi.org/10.5061/dryad.wwpzgmst6)”.

As you address these items, please take this last chance to review your reference list to ensure that it is complete and correct. If you have cited papers that have been retracted, please include the rationale for doing so in the manuscript text, or remove these references and replace them with relevant current references. Any changes to the reference list should be mentioned in the cover letter that accompanies your revised manuscript.

We have reviewed the reference list.
